# Warfarin prevented de novo portal vein thrombosis after transjugular intrahepatic portosystemic shunt: A retrospective study

**DOI:** 10.1097/MD.0000000000018737

**Published:** 2020-01-10

**Authors:** Linhao Zhang, Hui Huan, Huan Tong, Bo Wei, Zhidong Wang, Chao Liu, Hao Wu

**Affiliations:** a Department of Gastroenterology; b Laboratory of Gastroenterology and Hepatology, West China Hospital, Sichuan University; c Department of Gastroenterology, Hospital of Chengdu Office of People's Government of Tibetan autonomous Region, Sichuan, China.

**Keywords:** liver cirrhosis, portal vein thrombosis, retrospective study, transjugular intrahepatic portosystemic shunt, warfarin

## Abstract

Portal vein thrombosis (PVT) might impair the prognosis of cirrhotic patients. However, formation of de novo PVT after transjugular intrahepatic portosystemic shunt (TIPS) in cirrhotic patients without preexisting PVT was rarely reported. Moreover, it is not known whether warfarin is efficient in preventing de novo PVT after TIPS. The current study aimed to investigate retrospectively the incidence and location of de novo PVT, and preventive effects of warfarin on de novo PVT after TIPS for cirrhotic patients. Patients who received TIPS placement between March 1, 2015 and March 1, 2016 in our hospital were screened retrospectively. Patients without preexisting PVT before TIPS and those who were followed up for at least 12 months were included. There were 2 groups: 1 group received warfarin (warfarin group) post-TIPS, while another group (control group) did not receive prophylactic drug to prevent PVT. Their baseline characteristics and follow-up data were retrieved. The occurrence of PVT, adverse events due to warfarin, difference in stent patency and clinical complications such as stent dysfunction, hepatic encephalopathy, mortality, liver cancer, variceal bleeding, infection, and liver failure, and results of follow-up biochemical examination were compared. Eighty-three patients without preexisting PVT were included. There were 56 patients in the control group and 27 in the warfarin group. The incidence of PVT in the warfarin group was 14.8% (4/27), whereas the incidence in the control group was 42.9% (24/56, *P* = .013). The location of de novo PVT was mainly at left portal vein. Adverse events due to warfarin was mostly mild, such as hemorrhinia and gingival hemorrhage. No significant difference regarding to stent patency and clinical complications between the 2 groups was found. At 24-month after-TIPS, for the remaining patients in both groups, the total bilirubin was significantly increased while the red blood cell count was significantly decreased in control group compared with those in warfarin group (*P* < .05). PVT could commonly occur after TIPS in patients without preexisting PVT. Warfarin could prevent PVT in these patients, and might improve patient's liver function.

## 1 Introduction

Portal vein thrombosis (PVT) is commonly known as the thrombus formed within the portal vein trunk and intrahepatic portal branches.^[1]^ It was suggested that PVT occurs in approximately 10% to 30% of cirrhotic patients.^[2–4]^ Though the effect of PVT on prognosis of cirrhotic patients is controversial,^[5–8]^ treatment of PVT still might be able to reduce mortality and complications in these patients.^[1,9]^

Anticoagulation therapy has been recommended to treat PVT. A meta-analysis concluded that anticoagulant therapy could facilitate recanalization in almost 67% of patients without lethal complications in the context of liver cirrhosis.^[10]^ Additionally, transjugular intrahepatic portosystemic shunt (TIPS) is also suggested as the treatment for PVT in liver cirrhosis,^[1,11]^ and several studies demonstrated that TIPS placement alone could prevent PVT progression and/or achieve recanalization remarkably.^[5,12,13]^

On the other hand, according to our clinical observation and some recently published studies, de novo PVT might develop after TIPS placement.^[6,14]^ Post-TIPS administration of warfarin might be considered in these patients to prevent de novo PVT. However, to date, the incidence of de novo PVT after TIPS is rarely reported. The data on the preventive effects of warfarin on de novo PVT occurrence after TIPS placement is also limited. Therefore, we performed this retrospective study mainly aiming to investigate the incidence and location of de novo PVT, and preventive effects of warfarin on de novo PVT after TIPS for cirrhotic patients.

## 2 Methods

### 2.1 Patients

We retrospectively screened the data of consecutive patients with liver cirrhosis who received elective TIPS placement between March 1, 2015 and March 1, 2016 by our research team in our hospital. Liver cirrhosis was diagnosed based on the following items: clinical manifestations (history of liver diseases, liver function tests, and/or portal hypertension-related complications), imaging (ultrasound and computed tomography (CT)), and/or liver biopsy. The inclusion criteria were as follows:

(1) aged from 18 to 75 years old,
(2) indications of TIPS were refractory ascites and/or variceal bleeding,
(3) no evidence of preexisting PVT before TIPS (determined by contrast-enhanced CT),
(4) duration of follow-up was no less than 12 months.

The exclusion criteria were as follows:

(1) previous TIPS placement or liver transplantation,
(2) recent history (<3 months) of using anticoagulants or antiplatelet drugs before TIPS,
(3) platelet count  <20,000/mm^3^ or international normalized ratio (INR) >2 before TIPS,
(4) a malignancy and/or myeloproliferative disorder before TIPS,
(5) Child-Pugh score >13 before TIPS,
(6) complicated with heart failure,
(7) complicated with renal dysfunction.

**Figure 1:**
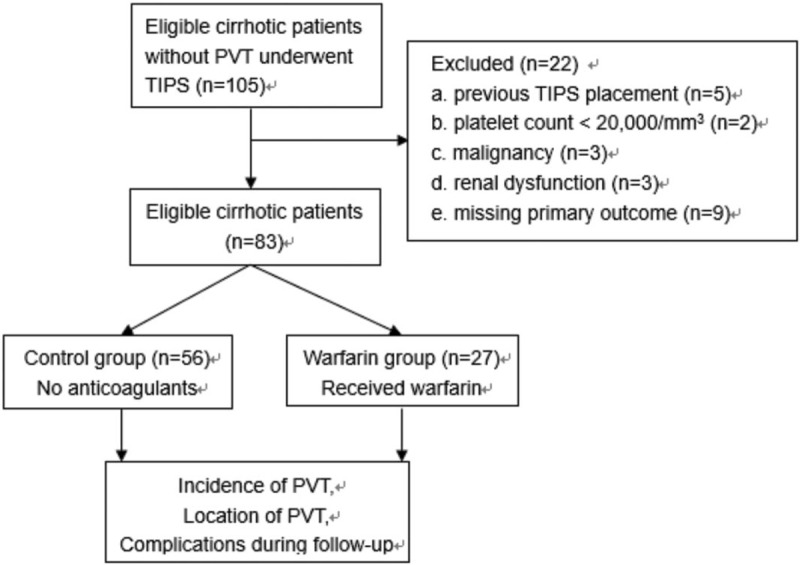
Flow chart. PVT = portal vein thrombosis, TIPS = transjugular intrahepatic portosystemic shunt.

In total, 105 cirrhotic patients met the inclusion criteria during the time period. 22 patients were excluded (Fig. 1). Among them, 5 was due to previous TIPS placement, 2 due to low platelet count, 3 due to malignancy, 3 due to renal dysfunction, and 9 due to missing primary outcome. Thus, 83 patients were finally included. Baseline characteristics of patients on admission were retrieved from their medical records, which included gender, age, etiology of liver cirrhosis, complications of liver cirrhosis, Child-Pugh score and class, model for the end-stage liver diseases (MELD) score, and related laboratory results (Table 1). This study conformed to the principles of Declaration of Helsinki for medical research and ethical approval was obtained from institutional review board of our hospital (No. 2019-298). The study was registered at the Chinese Clinical Trial Registry Center (ChiCTR1900021817). Written informed consent was exempted due to the retrospective nature. These is no subject overlap with any previous studies.

**Table 1:**
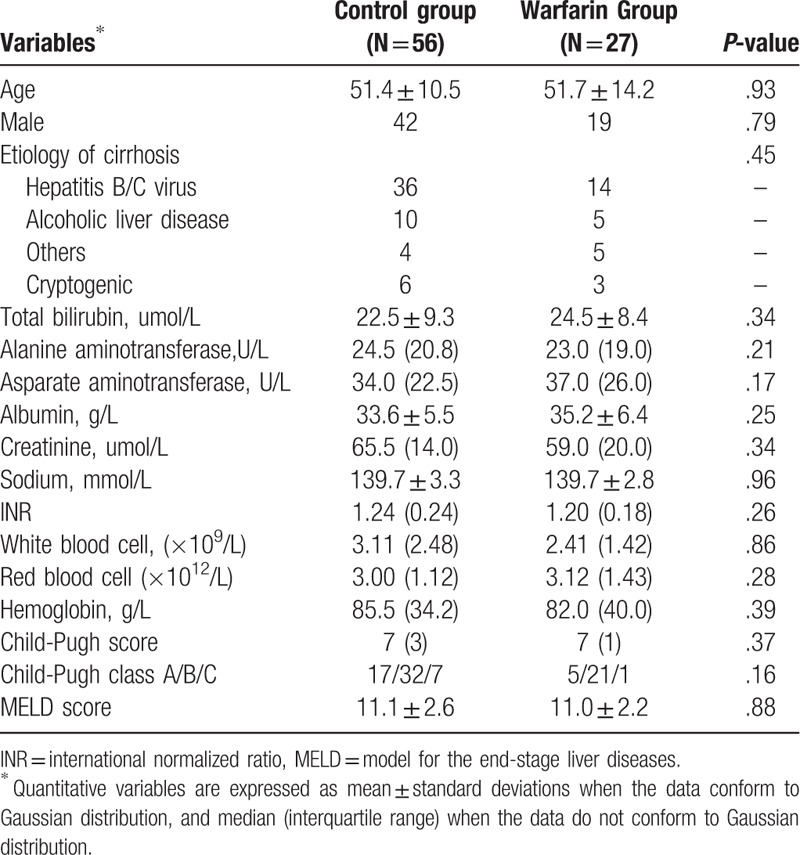
Baseline characteristics.

### 2.2 TIPS procedure

All TIPS were performed by well-trained practitioners, with a right internal jugular vein approach under local anesthesia as previously stated.^[15]^ After the puncture into hepatic vein with Rupus-100 (Cook Incorporated, Bloomington, IN), portal vein was accessed under X-ray guidance. Then, portography was performed and a balloon catheter (PowerFlex, Johnson & Johnson, Miami, FL) was dilated to create the intrahepatic tract. Finally, covered stents (Fluency Plus, Bard, Murray Hill, NJ) of 8 to 10 mm were placed and portal vein patency was confirmed by portography again afterwards. TIPS creation was confirmed to be technically successful by any item as follows: hepatic venous pressure gradient <12 mm Hg, or a hepatic venous pressure gradient decrement of >50% after stent implantation. In addition, 0.3 mL (3000 anti-Xa IU) of enoxaparin (Clexane; Sanofi Aventis, Maisons-Alfort, France) was empirically administered during the procedure.

### 2.3 Study design

Based on our previous clinical observations, we routinely explained the possibility of developing de novo PVT after TIPS placement to our patients in clinical practice. Moreover, since warfarin was proved to be safe in treating PVT in liver cirrhosis,^[15]^ we also recommended our patients to receive warfarin in order to prevent de novo PVT. In this way, some of the patients agreed to receive warfarin after TIPS. After reviewing clinical records, patients were divided into 2 groups. Detailed, 27 patients from warfarin group received warfarin after TIPS placement (initial dose 1.25–2.50 mg daily, which was adjusted sequentially until achieving the target INR of 2–3), while 56 patients in another group (control group) did not receive any prophylactic drugs to prevent de novo PVT after TIPS placement. During the out-patient follow-up, color doppler ultrasound was performed by experienced sonographers to identify PVT and stent patency. When PVT was identified by ultrasound, CT was recommended for further confirmation. PVT was defined as the presence of solid material in the lumen of portal vein and/or its tributaries. In the first 12-month post-TIPS, ultrasound was routinely performed at 1 week, 1 month, 3 months, 6 months, and 12 months; then, ultrasound was performed at least once in every 6 months. Biochemical examination was done and complications including gastrointestinal bleeding, hepatic encephalopathy, hepatorenal syndrome, spontaneous peritonitis, any episodes of infection, and liver decompensation were also recorded. The follow-up period began when TIPS was performed and ended with death, until the last ultrasound evaluation, or 36 months post-TIPS (depending on which came first).

The primary outcomes were the incidence and location of PVT after TIPS. The secondary outcomes were mortality, incidence of complications (eg, gastrointestinal bleeding and hepatic encephalopathy), and the difference in biochemical results after TIPS.

### 2.4 Statistical analysis

Data were compared between the control group and the warfarin group. Quantitative variables are expressed as mean ± standard deviations when the data conform to Gaussian distribution, and median (interquartile range) when the data do not conform to Gaussian distribution. Categorical variables are shown as number (percentage). Student *t* test, Mann–Whitney *U* test, Chi-square test, and Fisher exact test were done to compare quantitative and qualitative variables between groups, where applicable. Occurrences of PVT were described by Kaplan–Meier method and compared by the Log-rank test. Cox regression was used to analyze the factors in association with the occurrence of PVT in both groups. SPSS version 19.0 software (SPSS, Chicago, IL) was used for analysis. A *P* value <.05 was considered statistically significant.

## 3 Results

### 3.1 Patients

In total, 105 cirrhotic patients met the inclusion criteria during the time period. 22 patients were excluded. Thus, 83 patients were finally included into this study (Fig. 1). Among them, 56 did not receive anticoagulants (control group) and the rest 27 patients received warfarin (warfarin group). Their baseline characteristics were summarized (Table 1). There were no significant differences in age, gender, etiology of liver cirrhosis, laboratory results, Child-Pugh score, Child-Pugh class, and MELD score between the 2 groups (Table 1).

### 3.2 Follow-up and occurrence of PVT after TIPS

The mean follow-up period was 23.8 ± 9.9 months in the warfarin group and 25.0 ± 10.4 months in the control group (*P* = .62). Among the patients in warfarin group, 3 (11.1%) occasionally had INR value more than 3.00 (ranging from 3.44 – 5.28) during follow-up. All other patients were compliant and achieved target INR value in most of the coagulation test during follow-up. At 12-month after TIPS, the INR of patients in warfarin group was 2.50 (0.44).

**Figure 2:**
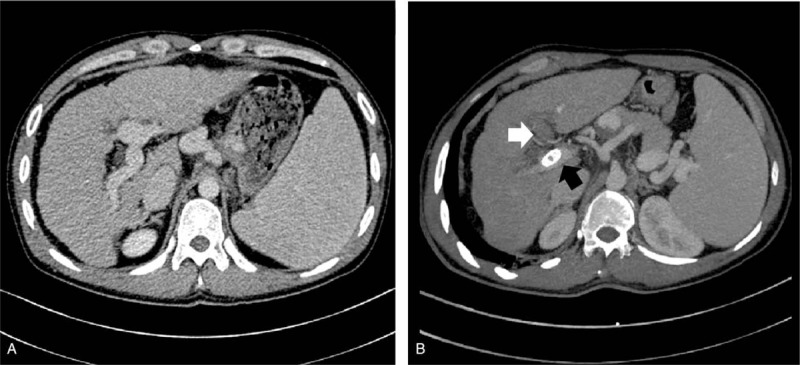
Typical de novo PVT found after TIPS in a patient from control group by computed tomography (CT). No PVT was observed before shunt creation (A). One month after TIPS, thrombosis of left portal branch was confirmed by contrast-enhanced CT after reconstruction (B). The black arrow indicates the contrast filling in right portal branch, while the white arrow indicates absence of contrast in left portal branch.

**Figure 3:**
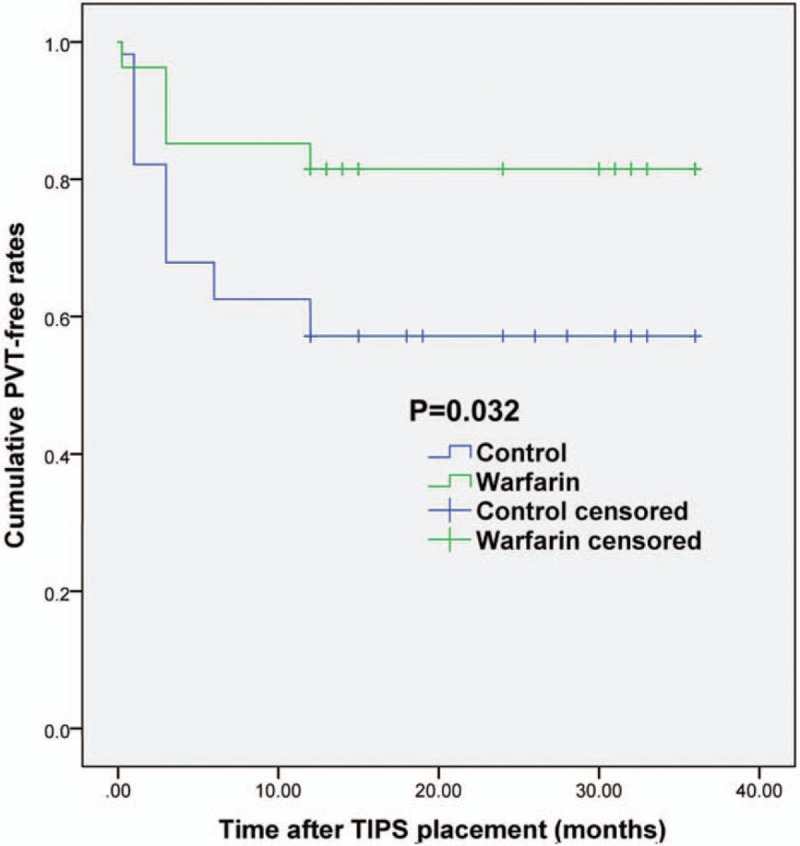
Kaplan–Meier curves. Cumulative PVT-free rates in the control group and warfarin group after TIPS.

A typical de novo PVT found post shunt creation after confirmation by CT was shown in Figure 2. PVT was found in 24 patients (42.9%) from control group, but only 4 patients (14.8%) in warfarin group had PVT (*χ*^2^ = 6.408, *P* = .013; Table 2). All the de novo PVT occurred within 12 months post-TIPS. The cumulative PVT free rates at 1 week, 1 month, 3 months, 6 months, and 12 months in the control group were 98.2%, 82.1%, 67.9%, 62.5%, and 57.1%, respectively (Fig. 3). By comparison, the cumulative PVT-free rates at 1 week, 1 month, 3 months, 6 months, and 12 months in the warfarin group were 96.3%, 96.3%, 85.2%, 85.2%, and 85.2%, respectively (*χ*^2^ = 4.582, *P* = .032, Fig. 3).

**Table 2:**
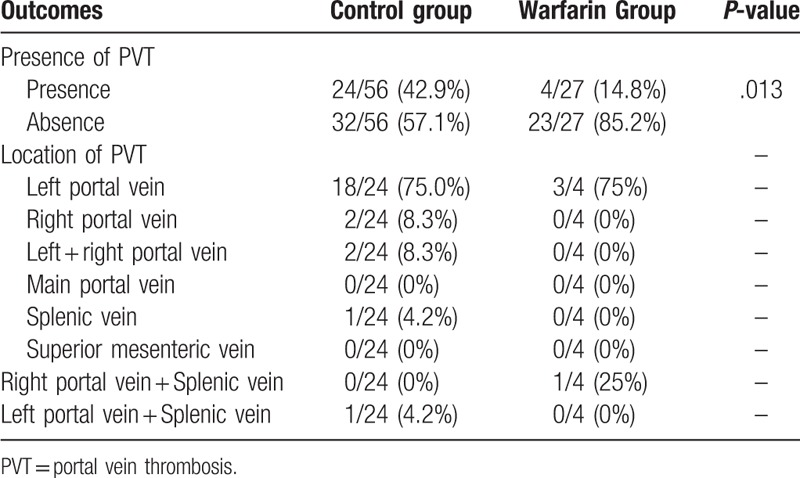
Primary outcomes.

In terms of the location of PVT, in the warfarin group, 3 and 1 patients had left PVT, and right portal vein plus splenic vein thrombosis, respectively. As for the control group, 2, 18, 2, 1, and 1 patients had right PVT, left PVT, left plus right PVT, splenic vein thrombosis, and left portal vein plus splenic vein thrombosis, respectively. Intriguingly, thrombosis of superior mesenteric vein or main portal vein was not observed and thrombosis of left portal vein was the most common type of PVT after TIPS.

Among the selected factors (use of warfarin, age, gender, etiology, total bilirubin, alanine transaminase, aspartate transaminase, albumin, creatinine, sodium, INR, red blood cell, hemoglobin, white blood cell, platelet, Child-Pugh score, Child-Pugh class, and MELD score), both univariate and multivariate Cox regressions found that only the use of warfarin could independently reduce the risk of formation of de novo PVT (*χ*^2^ = 4.771, *P* = .029, hazard ratio 0.307, 95% confidence interval 0.106–0.886; Table 3).

**Table 3:**
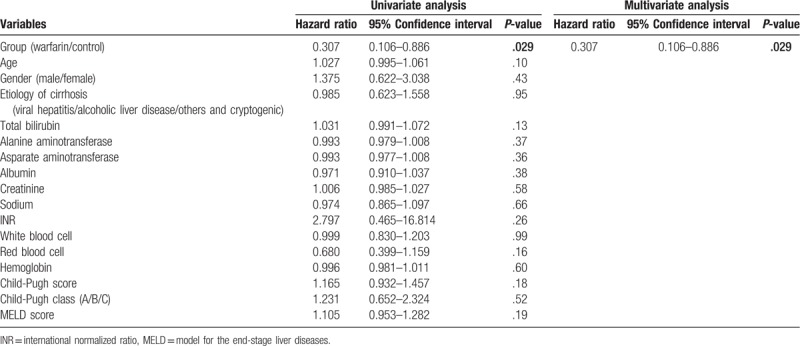
Cox regression: univariate and multivariate analyses of baseline factors associated with portal vein thrombosis formation.

### 3.3 Adverse events of warfarin

In the warfarin group, no severe adverse event due to warfarin was observed. Among them, common events were occasional hemorrhinia (7 patients, 25.9%) and gingival hemorrhage (5 patients, 18.5%), which usually happened when patients just started taking warfarin. These events were mild and tolerable, and would not exacerbate without decreasing warfarin dosage or applying other management. After taking warfarin for 6 months, 1 patient found scattered purpura in the skin of lower limbs and body trunk, and another was suspected to have hematemesis once with mild blood loss (it was stopped spontaneously before esophagogastroduodenoscopy and colonoscopy evaluation and both examinations did not detect any obvious lesion in gut). Their conditions were stable and did not need further hospitalization. The symptoms did not recur after reducing warfarin dosage to 1.25 mg daily.

### 3.4 Other clinical outcomes

TIPS placement was successful in all patients. During follow-up, stent patency was achieved in 92.6% (25/27) patients in warfarin group, and the patency rate was 92.9% (52/56) in control group (*P* > .99).

After TIPS placement, at least 1 episode of overt hepatic encephalopathy developed in 16 patients (28.6%) in the control group and in 6 patients (22.2%) in the warfarin group (*P* = .60). Two patients from the control group suffered from multiple episodes of hepatic encephalopathy which was improved after hospitalization, while others were resolved after medical therapy. Moreover, 2 patients in the control group died during the follow-up period (1 was due to liver cancer 12 months after TIPS, and another was because of variceal bleeding resulted from stent dysfunction 31 months after TIPS), while no death was observed in the warfarin group (*P* > .99). One patient (3.7%) from the warfarin group and 4 (7.1%) from the control group was diagnosed with liver cancer (*P* > .99). In terms of gastrointestinal bleeding, 2 out of 27 patients (7.4%) from the warfarin group suffered from variceal bleeding due to shunt dysfunction, while 5 out of 56 patients (8.9%) from the control group was reported to have variceal bleeding. Among the 5 patients in the control group, 4 was due to shunt dysfunction and the remaining 1 was due to portal vein tumor thrombus resulted from liver cancer (*P* > .99). Furthermore, 1 patient from the control group and another from the warfarin group had infection during follow-up. One patient from the control group suffered from acute on chronic liver failure.

Twelve-month after TIPS placement, the difference of characteristics between the 2 groups was not significant (*P* > .05; Table 4), though the total bilirubin of control group seemed to be higher than that of warfarin group (43.0 [20.3] vs 36.9 [14.9]; *P* = .05). However, 24-month after TIPS placement, for the remaining patients (31 patients in control group and 14 patients in warfarin group), the difference in their total bilirubin level was significant (50.3 [29.8] in control group vs 31.5 [19.4] in warfarin group; *P* = .018; Table 5). Moreover, the red blood cell count in control group (3.81 ± 0.95 × 10^9^/L) was also significantly lower than that in the warfarin group (4.52 ± 0.91 × 10^9^/L) (*P* = .042; Table 5).

**Table 4:**
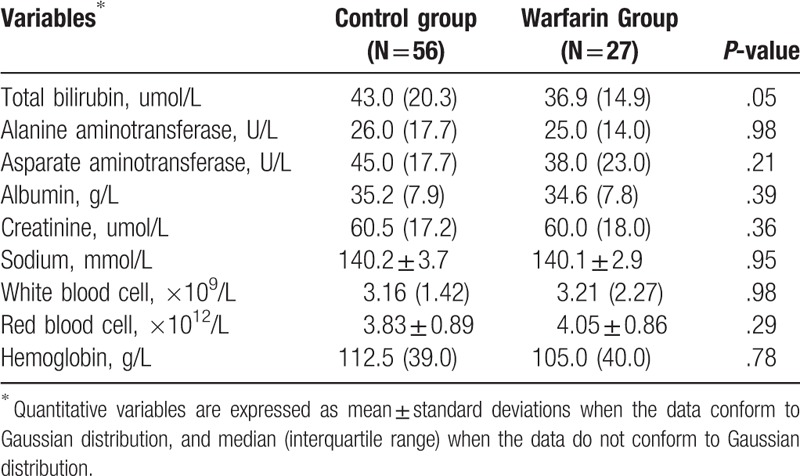
Follow-up data: 12-mo after transjugular intrahepatic portosystemic shunt placement.

**Table 5:**
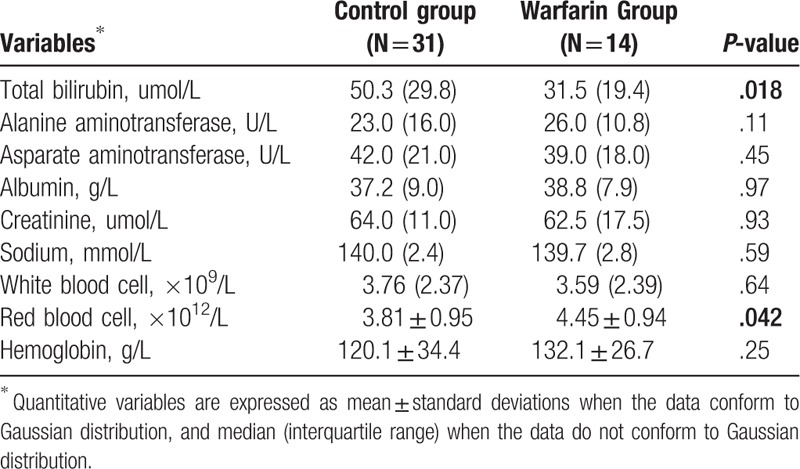
Follow-up data: 24-mo after transjugular intrahepatic portosystemic shunt placement.

## 4 Discussion

In the current study, it was found that for cirrhotic patients without history of PVT, TIPS placement could lead to de novo PVT, especially thrombosis of left portal vein (75% of PVT in both groups). More patients from the control group (approximately 43%) developed PVT during follow-up than that from the warfarin group (15%). This indicated that warfarin could reduce the incidence of de novo PVT after TIPS. Considering the potential detrimental effect of PVT on cirrhotic patients,^[5,16]^ these results suggested that warfarin should be taken into account to prevent PVT for patients with liver cirrhosis post-TIPS. To our knowledge, study using warfarin to prevent de novo PVT in cirrhotic patients post-TIPS was rarely reported.

It has been a consensus that TIPS could induce recanalization of PVT in patients with preexisting PVT.^[15,17]^ However, it was found that de novo PVT, especially the thrombosis of the left portal branch could occur after TIPS. Previously, Wan et al published the first paper concerning de novo PVT after TIPS,^[6,14]^ and they also observed PVT formation in patients who did not show preexisting PVT before TIPS. In their study, 26.7% of the patients had PVT during follow-up after TIPS and the main type of PVT post TIPS was also the thrombosis of the portal branch (11 out of 27 cases of PVT).^[6,14]^ However, a higher rate of PVT was observed and at 12th month, more than 40% of the control patients developed PVT. The thrombosis of the left portal branch was the main type of PVT in our patients (18 out of 24 cases in control patients and 3 out of 4 cases in warfarin group). This high rate of PVT after TIPS might be attributed to the fact that our control patients did not receive anticoagulant or antiplatelet therapy but the patients in Wan's study received aspirin or clopidogrel after TIPS.^[6,14]^ In this way, our data might be more accurate reflexing the real natural history of de novo PVT occurrence after TIPS procedure. However, due to the small sample size, this should be further evaluated in more studies.

**Figure 4:**
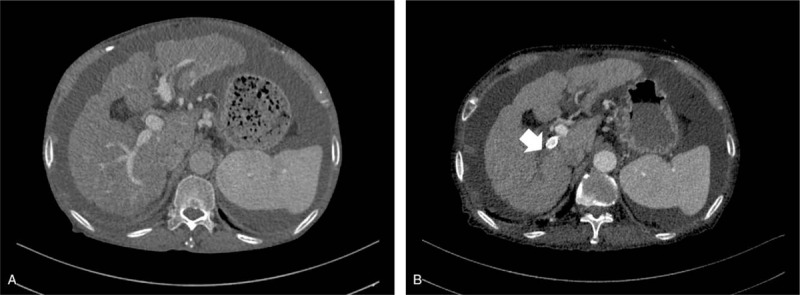
Typical de novo PVT found after TIPS using Viatorr stent in a patient by computed tomography (CT). No PVT was observed before shunt creation (A). 40 d after TIPS, thrombosis of right portal branch was confirmed by contrast-enhanced CT (B). The white arrow indicates absence of contrast in right portal branch.

The cause of PVT, especially the thrombosis of left portal branch, post-TIPS procedure was unknown. This might be due to the fact that most of the shunts were placed in the right portal branch, thus largely increasing its blood flow while the left portal vein might have less and slower bloodstream, facilitating the formation of thrombosis. Another potential cause might be the fact that we used Fluency stent, which does not have partially uncovered portion compared to Viatorr stent. Fluency stent was used because by the time the study was performed, Viatorr was not available in our hospital. However, we are currently using Viatorr instead of Fluency (starting from 2019), but we still identified de novo PVT after TIPS placement in a number of our patients (Fig. 4). Thus, we believe development of de novo PVT should be a common but previously neglected phenomenon after TIPS placement in liver cirrhosis, though its pathogenesis should be explored further.

Whatever the cause of PVT in these patients, its formation might be detrimental to their prognosis. During follow-up, it was found that the incidence of hepatic encephalopathy and the level of total bilirubin at 12-month post-TIPS was slightly though not significantly higher in the control group. Moreover, at 24-month after TIPS, for the remaining patients in both groups, we found total bilirubin was higher but red blood cell count was lower in control group than that in warfarin group. It has long been known that increase of total bilirubin is associated with poor prognosis in liver cirrhosis.^[18]^ Also, some study also suggested that decrease in red blood cell count could be a predictor of impaired liver function.^[19]^ Since incidence of PVT in control patients was higher, these results might indicate development of PVT might have negative impact on patients’ liver function. Moreover, when liver transplantation is needed, PVT might hamper the technique and contributes to posttransplant morbidity and mortality.^[20,21]^ Thus, solutions to cope with PVT after TIPS insertion are needed.

Anticoagulant has been used to treat PVT in cirrhotic patients, and a meta-analysis suggested that it could increase recanalization and reduce progression of PVT.^[22]^ Futhermore, recently Wan et al reported that after de novo PVT formation post-TIPS, warfarin could also effectively induce recanalization in about 50% of their patients within 1 year.^[6,14]^ In this study, warfarin was used to prevent PVT in cirrhotic patients after TIPS. It was identified that the incidence of PVT in warfarin group (15%) almost reduced by 3 times compared with that of control group (43%). This result is promising and considering the detrimental effects of PVT, we recommend that warfarin should be considered to prevent de novo PVT after TIPS procedure. By the way, it was interesting to observe similar stent patency rate in both groups. This actually was not surprising because covered stents were used and these stents per se guaranteed long period of stent patency, with or without post-TIPS anticoagulation, as evidenced by a previous study.^[15]^

Bleeding events are always the concern when anticoagulant is prescribed to patients with liver cirrhosis because liver cirrhosis has been linked to coagulopathy in the common belief. However, current evidences have suggested that coagulopathy might not truly reflect the reality in cirrhotic patients.^[23]^ For example, Lai et al found that the risk of intracerebral hemorrhage in cirrhotic patients was similar to that in control.^[24]^ Moreover, though low platelet count and increase in INR are evident in liver cirrhosis, recent findings indicate that traditional tests of prothrombin time and activated partial thromboplastin time could not detect the real coagulation balance in liver cirrhosis and hypercoagulation is actually the case in these patients.^[25]^ This is part of the rational to use anticoagulants such as warfarin in liver cirrhosis. In our study, it was found that warfarin did not induce severe complications in cirrhotic patients after TIPS procedure. Hemorrhinia and gingival bleeding were common but no extra treatment was needed. Though subcutaneous hemorrhage and hematemesis were observed in 2 patients, no severe consequence ensued after reducing the dose of warfarin. Consistently, other studies utilizing warfarin in patients with liver cirrhosis reported hemorrhinia and/or gingival hemorrhage as most common bleeding events, and gastrointestinal bleeding due to warfarin is usually rare.^[6,14,15]^ Thus, these results confirmed that warfarin is relatively safe in cirrhotic patients.

There are a number of limitations in this study. First, our inclusion criteria were relatively strict which required that the follow-up period was at least 12 months after TIPS. This resulted in a smaller sample size in our study. However, we think it guaranteed that most de novo PVT have been observed during follow-up. Second, we only observed the patients for at most 36 months. Longer observation period might be better but according to current results, longer observation might not alter the primary results because all de novo PVT occurred within 12-month post shunt creation. Third, for the patients in warfarin group, the prescription of warfarin was purely depended on patients’ willingness. In this way, selection bias could not be excluded and future study should, where possible, perform randomization. Forth, for patients in control group, we did not ask them to use warfarin after they developed PVT. This was because that they did not show obvious symptoms or signs due to PVT at least immediately after the presence of PVT. Thus, we currently do not know whether warfarin could treat these de novo PVT after TIPS placement, and future prospective study should evaluate this condition. Fifth, this was a retrospective study and the number of patients in both groups were not large. We understand that there were bias in the current study, and in this way, further studies recruiting more patients are needed.

In conclusion, warfarin was used to prevent de novo PVT in cirrhotic patients after TIPS creation in this study. PVT, especially thrombosis of left branch of portal vein, could commonly occur after shunt creation in patients without preexisting PVT prior to TIPS procedure. Warfarin could effectively prevent PVT in these patients and might improve patient's liver function.

## Acknowledgment

The authors are grateful to the statistical assistance from Yi-Long Chen (West China Biomedical Big Data Center, West China Hospital, Sichuan University, Chengdu, China).

## Author contributions

**Conceptualization:** Bo Wei, Hao Wu.

**Data curation:** Linhao Zhang, Hui Huan, Zhidong Wang, Hao Wu.

**Formal analysis:** Linhao Zhang, Hui Huan, Huan Tong, Hao Wu.

**Funding acquisition:** Linhao Zhang, Huan Tong, Hao Wu.

**Investigation:** Linhao Zhang, Bo Wei, Zhidong Wang.

**Methodology:** Linhao Zhang, Hui Huan, Huan Tong, Bo Wei, Zhidong Wang, Chao Liu.

**Resources:** Chao Liu, Hao Wu.

**Supervision:** Chao Liu, Hao Wu.

**Writing – original draft:** Linhao Zhang.

**Writing – review and editing:** Hui Huan, Huan Tong, Hao Wu.
